# Prior COVID-19 infection increases degenerated oocytes but does not affect IVF outcomes: a prospective cohort study

**DOI:** 10.3389/fendo.2025.1599771

**Published:** 2025-05-22

**Authors:** Huijun Chen, Hongxin Guo, Qi Zhao, Yuan Li, Ge Lin, Philipp Kalk, Berthold Hocher, Fei Gong

**Affiliations:** ^1^ Institute of Reproductive and Stem Cell Engineering, NHC Key Laboratory of Human Stem Cell and Reproductive Engineering, School of Basic Medical Science, Central South University, Changsha, Hunan, China; ^2^ Clinical Research Center for Reproduction and Genetics in Hunan Province, Reproductive and Genetic Hospital of CITIC-Xiangya, Changsha, Hunan, China; ^3^ Department of Nephrology, Charité Universitätsmedizin Berlin, Berlin, Germany; ^4^ Diaverum Renal Care Center, Diaverum MVZ Am Neuen Garten Standort Ludwigsfelde, Potsdam, Germany; ^5^ Fifth Department of Medicine (Nephrology/Endocrinology/Rheumatology/Pneumology), University Medical Centre Mannheim, University of Heidelberg, Mannheim, Germany; ^6^ IMD Institut für Medizinische Diagnostik Berlin-Potsdam GbR, Berlin, Germany

**Keywords:** post-COVID-19, oocyte quality, embryo quality, pregnancy outcomes, time interval

## Abstract

**Background:**

The global health crisis of coronavirus disease 2019 (COVID-19) continues to impact people of all age groups worldwide. Recent studies increasingly support that COVID-19 infection may affect reproductive function, causing subfertility and infertility. It is a prospective observational cohort study conducted in the Reproductive and Genetic Hospital of CITIC-Xiangya. 781 women recovered from COVID-19 and 388 uninfected controls undergoing IVF treatment. All participants received standard IVF treatment. Oocyte and embryo quality parameters and pregnancy outcomes were analyzed. Primary outcomes were oocyte and embryo quality, secondary outcomes included clinical pregnancy rates.

**Results:**

The results show that the COVID-19 recovery group had a higher number of degenerated oocytes compared to controls (0.15 ± 0.40 *vs*. 0.10 ± 0.33, P=0.035). Regression analysis confirmed this association even after adjusting for confounding factors (Adjusted β: 0.065, 95% CI: 0.006-0.099, P=0.026). However, other parameters of oocyte and embryo quality were comparable between groups. No significant differences were observed in clinical pregnancy rate, implantation rate, early miscarriage rate, ectopic pregnancy rate, or ongoing pregnancy rate. The time interval between COVID-19 recovery and IVF treatment did not significantly affect outcomes.

**Conclusion:**

Our study indicates that prior COVID-19 infection is associated with a slightly increased risk of degenerated oocytes but does not significantly impact other IVF outcomes or subsequent pregnancy rates. The time interval post-infection does not appear to influence IVF outcomes, suggesting no need to delay treatment following COVID-19 recovery. These findings provide reassurance for women planning IVF after COVID-19 infection.

## Background

The global health crisis of coronavirus disease 2019 (COVID-19) continues to impact people of all age groups worldwide, which is caused by severe acute respiratory syndrome coronavirus 2 (SARS-CoV-2) infection (1). Recent studies increasingly support that COVID-19 infection may affect reproductive function, as the female reproductive system expresses relevant receptors such as angiotensin-converting enzyme 2 (ACE2), and transmembrane serine protease 2 (1). However, it is indicated that semen parameters seem unaffected by the pandemic, according to the recent study (2).

The COVID-19 infection detriments the pregnancy process (both maternal and fetal) in many cases. Several meta-analyses have shown a higher likelihood of preeclampsia, preterm birth, stillbirth, gestational diabetes, and low birth weight in pregnant individuals with SARS-CoV-2 infection compared to those without the infection (3–5).

Furthermore, many studies documented female reproductive function alteration after the COVID-19 infection. COVID-19 infection has been linked to menstrual abnormalities, such as prolonged cycles and altered flow volume (6). Others also stated a shortened or disordered menstrual cycle as well as an increased volume of menstruation (7). Additionally, emerging evidence suggests impacts on ovarian reserve, sex hormone levels, and endometrial receptivity (8). For instance, genes critical for endometrial receptivity are dysregulated post-infection (9), and pre-existing conditions like endometriosis may worsen (10). These systemic disruptions collectively raise concerns about potential subfertility.

In a small-sample-sized observational study, the proportion of top-quality embryos is decreased in women after COVID-19 infection (11). A slight decrease in the blastocyst formation rate in the case group is also recorded (12). However, Soha Albeitawi et al. found no difference in oocyte and embryo quality as well as pregnancy outcomes (13), which is consistent with the results of other researchers (12, 14). However, the opposite opinion shows that the clinical and ongoing pregnancy rates are significantly lower in frozen embryo transfer cycles of patients with past SARS-CoV-2 infection (15). Unfortunately, current evidence is hard to prove the impact of COVID-19 on *in-vitro* fertilization (IVF) outcomes due to the inconsistent results as well as the limited evidence provided by these relatively small-sample-sized studies as indicated by a recent meta-analysis (16). Hence, we carried out this prospective cohort study with larger sample size to investigate the impact of COVID-19 infection and the time interval on the IVF outcomes in women who receive ovarian stimulation.

## Materials and methods

### Ethics and written consent

A prospective observational cohort study was performed to explore the effect of post-COVID-19 and time intervals on women undergoing IVF treatment. This study was approved by the Ethics committee of the reproductive and genetic hospital of CITIC-Xiangya (approval number: LL-SC-2023-012) and written consent was obtained from all participating patients.

### Participants

Patients were screened during the consultation step and those who met the inclusive and exclusive criteria were eligible and enrolled for our study from April 2023 to December 2023. The study group comprises individuals who have recovered from COVID-19 after being infected, while the control group consists of individuals who remained uninfected by COVID-19.

Inclusion criteria were: (1) age between 20 and 45 years old, (2) received ovarian stimulation, (3) women infected with COVID-19 before the treatment and the infection was confirmed by nucleic acid testing and/or antigen testing were enrolled in the study group (4), women who never infected COVID-19 (confirmed by nucleic acid testing and/or antigen testing or never had similar COVID-19 infection symptoms such as fever, sore throat, running nose, etc.) were enrolled in the control group.

Exclusion criteria were as follows: (1) women with suspicious COVID-19 symptoms (such as fever, sore throat, back pain, headache, etc.) but did not confirm by nucleic acid testing and/or antigen testing, (2) oocyte donation, (3) intrauterine insemination, (4) oocyte cryopreservation, (5) other conditions not applicable for assisted reproductive technology.

### Calculation of sample size

We set up a matching ratio between the study and control groups as 2:1, due to a decrease in the proportion of eligible participants in the control group after December 2022, when the Chinese government released “a circular on further optimizing the COVID-19 response”. The hypothesized clinical pregnancy rate difference across both groups is 10%. With a test efficacy of 90% and a significance level (α) set at 0.05, the calculated enrollment requirements are 760 individuals for the study group and 380 for the control group. After factoring in a 5% dropout rate, the final targeted enrollment is set at 800 individuals for the study group and 400 for the control group, resulting in a total of 1200 participants.

### Ovarian stimulation protocol

Controlled ovarian stimulation was conducted using gonadotropin-releasing hormone (GnRH) agonist protocol, GnRH-antagonist protocol, progestin-primed ovarian stimulation protocol, and others such as mild stimulation, etc. The choice of protocol (GnRH-agonist, antagonist, PPOS, or mild stimulation) was tailored to patient-specific factors, per center guidelines. The initial gonadotropin dosage was primarily determined by female age, body mass index (BMI), including anti-Müllerian hormone (AMH), antral follicle count (AFC), and basal follicle-stimulating hormone (FSH) levels. Throughout the stimulation process, follicular development was monitored through transvaginal ultrasound and serum hormone measurements, with adjustments made to the gonadotropin dosage as needed. Ovulation was triggered by administering human chorionic gonadotropin (hCG) after confirming adequate follicle stimulation by ultrasound and hormone concentrations.

Patients were slated for oocyte retrieval 35–36 hours after hCG administration. The procedures for oocyte retrieval, oocyte and embryo culture, insemination, intracytoplasmic sperm injection (ICSI), and embryo transfer were determined by the standard practices of the center, which holds ISO 9001 Certification.

### Outcome measurement

The primary outcomes in the present study were: oocytes and embryo quality. The secondary outcomes were: clinical pregnancy outcomes. Degenerated oocytes are defined as those that retrieved oocytes have undergone deterioration or degradation, making them non-viable for fertilization. Pregnancy outcomes were assessed exclusively in fresh embryo transfer cycles to minimize confounding by cryopreservation effects. The clinical pregnancy was identified as the presence of gestational sac(s) exhibiting fetal heart activity through ultrasound in the fourth week following embryo transfer. The implantation rate was calculated by dividing the total number of embryos transferred by the number of sacs. Subsequently, miscarriage was characterized as the loss of intrauterine pregnancy after the confirmation of gestational sacs (17). Pregnancy outcomes were followed up to three months after embryo transfer.

### Data analysis

Statistical Package for Social Sciences for Windows, version 25.0 (SPSS Inc, Chicago, IL, USA) was used to perform data analyses. The flowchart was generated using Edraw Max, version 9.2 (Shenzhen, China), and graphs were created using GraphPad Prism 8 (GraphPad Software, San Diego, USA). Homogeneity of variance and normality of data were estimated using the Shapiro–Wilk, Kolmogorov-Smirnov, and Lilliefors tests, respectively. Values were expressed as means ± standard deviation, or frequency (%). A comparison of quantitative variables (also continuous variables) between groups was performed using the Kruskal-Wallis test or ANOVA according to the normality. Qualitative variables (also categorical variables) were compared by the Chi-square (χ2) test or Fisher’s exact test. Multivariate logistic regression analysis was performed to figure out the risk factors of pregnancy outcomes. Data were considered statistically significant with a two-sided P < 0.05.

## Results

A total of 1300 infertile women were recruited in our study. Finally, there were 781 women recovered from COVID-19 and 388 women uninfected with COVID-19, a total of 1169 persons enrolled. The reasons for other participants’ exclusion were detailed and stated in our flow chart (**Supplementary Figure S1**). As for embryo transfer, 215 post-COVID-19 and 113 control women were performed and all of them were followed by three months after embryo transfer to collect the data on pregnancy outcomes for further analysis. Reasons for embryo transfer cancellation are as follows: 1) ovarian hyperstimulation syndrome (n=36), 2) no oocytes (n=8), 3) no transferrable embryo (n=79), 4) preimplantation genetic test (n=299), 5) asynchrony in embryo and endometrium (n=311), 6) personal reasons (n=108).

There is no significant difference in participants’ demographic information between the two groups on many levels, such as age, infertility duration, BMI, AMH, and AFC, while the waist-to-hip ratio is slightly higher in controls. In addition, no significant difference is observed in the basal levels of FSH, luteinizing hormone (LH), estradiol (E2), and progesterone (P) between the two groups (**Table 1**). We also tested some endocrine markers to evaluate the impact of COVID-19 infection on the endocrine function. Interestedly, the fasting glucose is lower in the study group (5.18 (4.3, 5.43) *vs*. 5.24 (5.02, 5.52), P=0.001) while the free thyroxine (T4) is higher (0.99 (0.92, 1.09) *vs*. 0.97 (0.90, 1.07), P=0.044). No difference is observed in blood pressure, blood cells, and coagulation function (**Table 1**).

**Table 1:** Demographic information of participants.

	Women after COVID-19 infection (n=781)	Controls (n=388)	P value
Age (year)	32.00 (29.00, 36.00)	33.00 (30.00, 37.00)	0.058
Infertility			
Primary	33.67 (263/781)	32.22 (125/388)	0.769
Secondary	57.11 (446/781)	59.28 (230/388)	
Others	9.22 (72/781)	8.51 (3/388)	
Infertility duration (year)	3.00 (1.00, 4.00)	3.00 (2.00, 5.00)	0.062
BMI (kg/m2)	22.32 (20.33, 24.14)	22.60 (20.82, 24.56)	0.053
Waist-to-hip ratio	0.81 (0.77, 0.85)	0.82 (0.78, 0.87)	0.001
AMH (ng/ml)	2.81 (1.56, 4.85)	2.67 (1.36, 4.20)	0.095
AFC	21.00 (12.00, 32.00)	19.00 (10.00, 31.00)	0.273
Basal FSH (mIU/ml)	6.35 (5.22, 7.92)	6.53 (5.13, 7.96)	0.609
Basal LH (mIU/ml)	4.21 (2.69, 6.25)	4.43 (3.00, 6.28)	0.156
Basal E2 (pg/ml)	40.84 (29.93, 94.45)	40.20 (27.80, 78.64)	0.114
Basal P (ng/ml)	0.37 (0.19, 1.14)	0.34 (0.17, 0.96)	0.194
Fasting glucose (mmol/L)	5.18 (4.3, 5.43)	5.24 (5.02, 5.52)	0.001
Fasting insulin (μIU/mL)	7.63 (5.40, 20.50_	7.80 (5.90, 11.10)	0.081
HOMA-IR	1.77 (1.22,2.44)	1.73 (1.31, 2.52_	0.485
Free T3 (pg/ml)	2.82 (2.60, 3.07)	2.82 (2.61, 3.08)	0.670
Free T4 (ng/ml)	0.99 (0.92, 1.09)	0.97 (0.90, 1.07)	0.044
TSH (μIU/ml)	1.76 (1.21, 2.48)	1.80 (1.26, 2.55)	0.400
Systolic blood pressure (mmHg)	114.00 (105.00, 122.00)	115.00 (106.25, 122.00)	0.161
Diastolic blood pressure (mmHg)	73.00 (68.00, 80.00)	73.00 (67.00, 80.00)	0.827
WBC (x10^9/L)	6.10 (5.10, 7.40)	6.10 (5.20, 7.50)	0.470
RBC (x10^12/L)	4.45 (4.24, 4.69)	4.47 (4.23, 4.70)	0.713
HGB (g/L)	134.00 (128.00, 140.00)	135.00 (128.00, 141.00)	0.231
MCV (fl)	90.40 (87.80, 93.00)	90.80 (88.23, 93.20)	0.385
PLT (x10^9/L)	242.00 (206.00, 286.00)	243.50 (204.00, 277.00)	0.589
D-Dimer (mg/L)	0.24 (0.15, 0.36)	0.23 (0.15, 0.34)	0.382
APTT (s)	33.20 (30.95, 35.40)	32.85 (30.83, 35.20)	0.302
PT (s)	11.00 (10.60, 11.40)	11.00 (10.63, 11.40)	0.724
FIB (g/L)	2.80 (2.51, 3.15)	2.86 (2.50, 3.18)	0.489
TT (s)	14.00 (13.20, 14.80)	14.00 (13.20, 14.80)	0.863

BMI. body mass index; AMH, anti-Müllerian hormone; AFC, antral follicle count; FSH, follicle-stimulating hormone; LH, luteinizing hormone; E2, estradiol; P, progesterone; HOMA-IR, homeostatic model assessment of insulin resistance; T3, triiodothyronine; T4, thyroxine; TSH, thyroid-stimulating hormone; WBC, white blood cell count; RBC, red blood cell count; HGB, hemoglobin; MCV, mean corpuscular volume; PLT, platelet; APTT, activated partial thromboplastin time; PT, prothrombin time; FIB, fibrinogen; TT, thrombin time.

The proportion of women treated with different stimulation protocols between the two groups is similar. The dose of stimulation drugs, stimulation duration, and hCG for triggering, have no significant difference in whether the patients suffered from COVID-19 before or not. On hCG day, the levels of E2, P, LH, and follicles are alike in two groups. Besides, the oocyte and embryo quality are also parallel between the two groups, such as different stages of oocytes, the number of 2 pronuclei (2PN) zygotes, fertilization rate, cleavage embryos, the number of day 3 good quality embryo, blastocyst formation rate, and the number of good-quality blastocyst. However, the number of degenerated oocytes is higher in the study group (0.15 ± 0.40 *vs*. 0.10 ± 0.33, P=0.035) (**Table 2**). Women after COVID-19 infection also had a higher proportion of ICSI and half IVF+half ICSI treatment (**Table 2**). Further regression analysis shows that prior COVID-19 infection is positively related to the number of degenerated oocytes (Adjusted β: 0.063, 95% confidence interval (CI): 0.004 -0.097, P=0.032) (**Table 3A**). To adjust the confounding factors, multivariate regression analysis was applied, and it also shows the same correlation (Adjusted β: 0.065, 95% CI: (0.006, 0.099), P=0.026) (**Table 3B**). Additionally, we also evaluated confounding factors related with endocrine parameters. Surprisingly, prior COVID-19 is a protecting factor for fasting glucose (Adjusted β: -0.066, 95% CI: (-0.123, -0.009), P=0.023) while it is not related to free T4 level (Adjusted β: 0.005, 95% CI: (-0.668, 0.783), P=0.876) (**Table 3C**).

**Table 2:** Ovarian stimulation outcomes.

	Women after COVID-19 infection (n=781)	Controls (n=388)	P value
Protocol (%)			
GnRH-agonist protocol	39.95 (312/781)	41.39 (161/389)	0.765
GnRH-antagonist protocol	39.05 (305/781)	35.99 (140/389)	
PPOS	14.47 (113/781)	15.94 (62/389)	
Others*	6.53 (51/781)	6.43 (25/389)	
Gn dosage/[IU]]	2287.50 (1625.00, 3000.00)	2281.25 (1700.00, 2896.88)	0.970
Gn duration/[day]	10.00 (9.00, 12.00)	10.00 (9.00, 12.00)	0.886
E2 on hCG day/[pg/ml]	2880.00 (1660.25, 4260.00)	2957.50 (1625.00, 4257.50)	0.759
P on hCG day/[ng/ml]	0.69 (0.47, 1.03)	0.65 (0.41, 1.03)	0.166
LH on hCG day/[mIU/ml]	2.30 (1.54, 3.90)	2.48 (1.67, 4.12)	0.053
hCG dosage for triggering (IU)	5000.00 (5000.00, 6000.00)	5000.00 (5000.00, 6000.00)	0.840
Follicles on hCG day	11.00 (7.00, 14.00)	11.00 (6.00, 14.00)	0.832
No. of oocytes retrieved	10.00 (6.00, 14.00)	10.00 (6.00, 14.00)	0.455
MII oocytes	9.00 (5.00, 13.00)	8.00 (5.00, 12.00)	0.410
MI oocytes	0.76 ± 1.39	0.89 ± 1.66	0.372
GV oocytes	0.69 ± 1.35	0.60 ± 1.43	0.065
Degenerated oocytes	0.15 ± 0.40	0.10 ± 0.33	0.035
No. of 2PN zygotes	6.00 (3.00, 9.00)	6.00 (3.00, 9.00)	0.292
Fertilization methods (%)			
IVF	49.55 (387/781)	59.54 (231/388)	0.004
ICSI	44.43 (347/781)	36.34 (141/388)	
IVF+ICSI	6.02 (47/781)	4.12 (16/388)	
Fertilization rate (%)	63.40 (50.00, 78.57)	62.50 (45.45, 78.57)	0.493
Cleavage embryos	7.00 (4.00, 11.00)	8.00 (4.00, 11.00)	0.776
The number of day 3 good quality embryos	3.00 (1.00, 6.00)	3.00 (1.00, 6.00)	0.624
Blastocyst formation rate (%)	41.79 (2078/4972)	41.24 (921/2233)	0.662
The number of good-quality blastocysts	0.33 ± 0.99	0.34 ± 0.98	0.779

GnRH, gonadotropin-releasing hormone; PPOS, progestin-primed ovarian stimulation; Gn, gonadotropin; LH, luteinizing hormone; E2, estradiol; P, progesterone; hCG, human chorionic gonadotropin; MII, metaphase II (reflects oocytes quality, only MII oocytes can be fertilized); MI, metaphase I; GV, germinal vesicle; 2PN, pronucleus; IVF, *in vitro* fertilization; ICSI, intracytoplasmic sperm injection.

Good quality embryo is defined as D3 embryo ≥7C-II and blastocyst ≥4BB while fair embryo is defined as D3 embryo <7C-II and blastocyst <4BB.

Data are given as medians (interquartile ranges), means ± standard deviation, or number (percentage).

*Others include mild stimulation, Gn stimulation, letrozole protocol, and natural cycle.

**Table 3A:** Regression analysis. Univariate regression analysis on the association between prior infection, time interval and IVF outcomes.

	COVID-19 infection				Time interval			
	Adjusted β/OR	95% CI		P value	Adjusted β/OR	95% CI		P value
Oocytes retrieved	0.024	-0.434	1.062	0.410	0.005	-0.010	0.011	0.890
MII oocytes	0.027	-0.351	0.977	0.355	0.021	-0.007	0.012	0.562
MI oocytes	-0.042	-0.315	0.047	0.148	-0.034	-0.003	0.001	0.348
GV oocytes	0.029	-0.083	0.252	0.323	-0.021	-0.003	0.002	0.563
Degenerated oocytes	0.063	0.004	0.097	0.032	-0.023	-0.001	0.001	0.522
2PN zygotes	0.028	-0.270	0.779	0.342	0.001	-0.007	0.007	0.992
Cleavage embryos	0.008	0.781	-0.520	0.691	0.029	-0.005	0.012	0.414
Day 3 good-quality embryo	0.011	-0.358	0.533	0.700	0.008	-0.006	0.007	0.828
Good-quality blastocyst	-0.003	-0.126	0.114	0.922	-0.052	-0.003	0.001	0.145
Clinical pregnancy	0.826	0.616	1.109	0.204	0.998	0.993	1.002	0.309
Implantation	-0.029	-0.112	0.037	0.332	-0.025	-0.001	0.001	0.477
Ongoing pregnancy	0.880	0.650	1.190	0.406	0.999	0.994	1.003	0.564

Time interval refers to the days from COVID-19 infection to IVF treatment.

**Table 3B:** Multivariate regression analysis on the degenerated oocytes.

	Adjusted β	P value	95% CI of β	
			Lower bound	Upper bound
Constant		0.585	-0.297	0.168
Age	0.046	0.154	-0.001	0.008
BMI	-0.001	0.968	-0.008	0.007
AMH	0.057	0.203	-0.004	0.017
AFC	0.071	0.131	0.000	0.003
COVID-19 infection	0.065	0.026	0.006	0.099

**Table 3C:** Multivariate regression analysis on the endocrine parameters.

	Fasting glucose				Free T4			
	Adjusted β/OR	95% CI		P value	Adjusted β/OR	95% CI		P value
Constant		3.845	4.614	<0.001		0.346	10.151	0.036
Age	0.066	0.001	0.012	0.023	-0.025	-0.098	0.038	0.390
BMI	0.121	0.009	0.029	<0.001	0.006	-0.115	0.137	0.862
Waist to hip ratio	0.065	0.019	0.924	0.041	-0.035	-8.954	2.590	0.280
COVID-19 infection	-0.066	-0.123	-0.009	0.023	0.005	-0.668	0.783	0.876

Parameters related to glucose and thyroid function according to published literature were selected for the multivariate regression model.

OR, odd ratio; CI, confidential interval; MII, metaphase II (reflects oocytes quality, only MII oocytes can be fertilized); MI, metaphase I; GV, germinal vesicle; 2PN, pronucleus; AMH, anti-Müllerian hormone; AFC, antral follicle count; T4, thyroxine.

Good quality embryo is defined as D3 embryo ≥7C-II and blastocyst ≥4BB while fair embryo is defined as D3 embryo <7C-II and blastocyst <4BB;

In women who received embryo transfer, there is no difference in the endometrial thickness before transfer, the number of embryos transferred, and the number of good-quality embryos transferred (**Table 2**). Similarly, no difference is observed in the pregnancy outcomes such as clinical pregnancy rate, implantation rate, early miscarriage rate, ectopic pregnancy rate, and ongoing pregnancy rate (**Table 4**). Further regression analysis also shows that COVID-19 infection or not is not related to clinical pregnancy, embryo implantation, and ongoing pregnancy (**Table 3**).

**Table 4:** Pregnancy outcomes.

	Women after COVID-19 infection (n=215)	Controls (n=113)	P value
Endometrial thickness (mm)	12.60 (11.15, 13.80)	12.70 (11.60, 14.00)	0.664
The number of embryos transferred	2.00 (1.00, 2.00)	2.00 (1.00, 2.00)	0.810
Good-quality embryo transferred	1.00 (1.00, 2.00)	1.00 (1.00, 2.00)	0.166
Clinical pregnancy rate (%)	72.56 (156/215)	79.65 (90/113)	0.159
Implantation rate (%)	82.44 (216/262)	78.08 (114/146)	0.283
Early miscarriage rate (%)	2.79 (6/215)	5.31 (6/113)	0.226
Ectopic pregnancy rate (%)	0.47 (1/215)	1.77 (2/113)	0.274
Ongoing pregnancy rate (%)	69.30 (149/215)	72.57 (82/113)	0.538

Our participants are divided into four groups according to the time interval from COVID-19 infection to the IVF/ICSI treatment to illustrate whether recovery time plays a role in IVF/ICSI performance. Group 1 represents recovery time within 60 days (n=72), Group 2 represents recovery time between 60–120 days (n=557), group 3 is for recovery time between 120–180 days (n=107), and group 4 is over 180 days (n=45). The blastocyst formation rate differs among these groups, either compared with or without controls, but with no trend (**Table 5**). However, no difference was observed in oocyte and embryo quality as well as pregnancy outcomes in women experiencing different time intervals (**Tables 3**, **5**). Notably, there is an obvious decrease in implantation rate in women after COVID-19 infection, compared to controls (P=0.005) (**Table 5**).

**Table 5:** IVF and pregnancy outcomes in different time intervals after COVID-19 infection to IVF/ICSI treatment.

IVF outcomes	Group 1 (n=72)	Group 2 (n=557)	Group 3 (n=107)	Group 4 (n=45)	Controls (n=389)	P value^a^	P value^b^
Follicles on hCG day	11.00 (7.25, 13.75)	11.00 (7.00, 14.00)	11.00 (6.00, 14.00)	10.00 (7.00, 13.00)	11.00 (6.00, 14.00)	0.955	0.986
No. of oocytes retrieved	10.00 (7.00, 14.75)	10.00 (6.00, 14.00)	11.00 (6.00, 14.00)	11.00 (6.00, 14.50)	10.00 (6.00, 14.00)	0.947	0.922
MII oocytes	8.50 (5.00, 13.00)	9.00 (5.00, 13.00)	9.00 (5.00, 12.00)	9.00 (5.50, 12.50)	8.00 (5.00, 12.00)	0.861	0.842
MI oocytes	0.76 ± 1.20	0.79 ± 1.50	0.67 ± 1.09	0.58 ± 0.87	0.89 ± 1.66	0.937	0.877
GV oocytes	0.72 ± 1.19	0.67 ± 1.30	0.79 ± 1.71	0.58 ± 1.27	0.60 ± 1.43	0.467	0.188
Degenerated oocytes	0.22 ± 0.51	0.14 ± 0.38	0.17 ± 0.45	0.13 ± 0.34	0.10 ± 0.33	0.698	0.198
No. of 2PN zygotes	6.00 (3.25, 9.00)	6.00 (3.00, 9.00)	6.00 (3.00, 10.00)	6.00 (3.50, 9.00)	6.00 (3.00, 9.00)	0.868	0.766
Fertilization rate (%)	61.11 (50.00, 77.78)	63.82 (47.83, 79.38)	60.77 (50.00, 80.00)	61.54 (51.00, 75.00)	62.50 (45.45, 78.57)	0.957	0.939
Cleavage embryos	7.00 (4.00, 11.75)	7.00 (4.00, 11.00)	7.00 (4.00, 11.00)	8.00 (4.50, 11.50)	8.00 (4.00, 11.00)	0.623	0.768
The number of day 3 good quality embryo	4.00 (1.00, 6.00)	3.00 (1.00, 6.00)	3.00 (1.00, 6.00)	4.00 (1.00, 6.00)	3.00 (1.00, 6.00)	0.834	0.901
Blastocyst formation rate (%)	37.91 (160/422)	43.72 (1569/3589)	36.81 (247/671)	41.60 (109/262)	41.24 (921/2233)	<0.001	0.004
The number of good-quality blastocysts	0.24 ± 0.70	0.37 ± 1.06	0.21 ± 0.74	0.27 ± 0.89	0.34 ± 0.98	0.242	0.368

Pregnancy outcomes	Group 1 (n=23)	Group 2 (n=158)	Group 3 (n=23)	Group 4 (n=11)	Controls (n=113)	P value^a^	P value^b^
Clinical pregnancy rate (%)	69.57 (16/23)	72.15 (114/158)	78.26 (18/23)	72.73 (8/11)	79.65 (90/113)	0.917	0.638
Implantation rate (%)	64.86 (24/37)	59.77 (156/261)	69.44 (25/36)	64.71 (11/17)	78.08 (114/146)	0.666	0.005
Early miscarriage rate (%)	4.35 (1/23)	3.16 (5/158)	0	0	5.31 (6/113)	0.536	0.486
Ectopic pregnancy rate (%)	4.35 (1/23)	0	0	0	1.77 (2/113)	0.211	0.214
Ongoing pregnancy rate (%)	60.87 (14/23)	68.99 (109/158)	78.26 (18/23)	72.73 (8/11)	72.57 (82/113)	0.630	0.715

hCG, human chorionic gonadotropin; MII, metaphase II (reflects oocytes quality, only MII oocytes can be fertilized); MI, metaphase I; GV, germinal vesicle; 2PN, pronucleus; IVF, *in vitro* fertilization; ICSI, intracytoplasmic sperm injection.

Good quality embryo is defined as D3 embryo ≥7C-II and blastocyst ≥4BB while fair embryo is defined as D3 embryo <7C-II and blastocyst <4BB.

Data are given as medians (interquartile ranges), means ± standard deviation, or number (percentage).

Group 1: recovery time within 60 days.

Group 2: recovery time between 60–120 days.

Group 3: recovery time between 120–180 days.

Group 4: over 180 days.

a comparison among groups 1-4.

b comparison among all the 5 groups.

There are no differences in ovarian reserve factors (such as age, AMH, AFC and so on), which influence IVF outcomes, among the four groups.

## Discussion

### Main findings

In this prospective cohort study, we investigated the impact of prior COVID-19 infection on IVF outcomes. Our results indicate that while prior COVID-19 infection is associated with an increase in the number of degenerated oocytes, it does not significantly affect embryo quality or pregnancy outcomes. Additionally, the time interval between COVID-19 recovery and IVF treatment did not influence the outcomes, suggesting no need to delay IVF treatment following recovery from COVID-19 infection.

### Interpretation

The observed increase in degenerated oocytes among women with prior COVID-19 infection aligns with the known effects of SARS-CoV-2 on ovarian function (8) (18). SARS-CoV-2 utilizes the ACE2 receptor, which is abundantly expressed in ovarian tissue, potentially disrupting normal ovarian physiology (1). The downregulation of ACE2 by SARS-CoV-2 can impair ovarian function, as ACE2 plays a critical role in follicular development and oocyte maturation (19). However, despite this increase in degenerated oocytes, our study did not find a corresponding negative impact on embryo quality or pregnancy outcomes. There are some potential reasons could give an explanation to this result. First, we chose the good-quality embryos for transferring in the daily practice, suggesting that while there were more degenerated oocytes, there were still enough good quality oocytes and resulting embryos to use for transfer, and the increased rate of degenerated oocytes would not necessarily impact pregnancy rates. Secondly, the study found that other measures of oocyte and embryo quality such as the number of mature (MII) oocytes, fertilization rates, and numbers of good quality embryos/blastocysts were comparable between the COVID-19 recovery group and controls, indicating that the overall oocyte and embryo quality was most likely unaffected. Moreover, while statistically significant, the absolute difference in degenerated oocytes was relatively small (0.15 ± 0.40 vs. 0.10 ± 0.33). This small difference may not have been large enough to affect overall IVF outcomes. Finally, the study may not have had sufficient statistical power to detect small differences in pregnancy outcomes, particularly given the smaller sample size for fresh embryo transfers. In summary, while prior COVID-19 infection was associated with a slight increase in degenerated oocytes, this did not translate to poorer embryo quality or pregnancy outcomes, most likely due to the selection of good quality embryos for transfer and the overall comparable quality of viable oocytes and resulting embryos between groups. Our findings are consistent with previous studies showing no significant differences in IVF outcomes between women with and without prior COVID-19 infection (20, 21). For instance, Albeitawi et al. (13)and Youngster et al. (12, 14) reported similar results, reinforcing the notion that prior COVID-19 infection does not detrimentally affect IVF success rates.

### Impact on endocrine function

We observed significant effects on fasting blood glucose and free T4. While statistically significant, the effect sizes were minimal. For fasting glucose, the adjusted difference (−0.06 mmol/L) is far below thresholds for clinical relevance in glucose metabolism (e.g., diabetes diagnosis requires ≥7.0 mmol/L). This suggests incidental variation rather than pathology. On the other hand, for free T4, the slight increase (0.02 ng/mL) falls within normal physiological ranges and lacks correlation with TSH changes, indicating no thyroid dysfunction. We speculate these subtle shifts may reflect transient inflammatory or metabolic adaptations post-COVID-19, but they do not appear to impact reproductive outcomes.

### Coagulation and hematologic effects

Additionally, studies also reported that COVID-19 leads to coagulopathy as it directly induces the production of endogenous chemical substances that promote the alteration of vascular hemostasis (22). A higher D-dimer level, prothrombin time, fibrinogen, and thrombin time were reported in studies (23, 24). Quantitative hematologic abnormalities are also described in some studies including lymphocytopenia, neutrophilia, eosinopenia, and mild thrombocytopenia (24, 25). While certain changes returned to normal values after recovery, others endured for months, illustrating the lasting impact of COVID-19 on the body (26). Contrary to some reports of COVID-19-induced coagulopathy and hematologic abnormalities, we did not observe significant changes in coagulation function or blood cell counts among our participants. This discrepancy could be attributed to the recovery period before IVF treatment or the relatively mild nature of COVID-19 cases in our cohort.

### Timing of IVF treatment post-COVID-19

Women who recovered from COVID-19 and received frozen embryo transfer within 60 days exhibited a significantly lower pregnancy rate than controls. Conversely, when the time interval was above 60 days, the difference disappeared (15). To ensure the recruitment of healthy gametes not exposed to COVID-19 during their development, it is recommended for infertile women to delay IVF treatment for at least 3 months (the duration of folliculogenesis and spermatogenesis) after recovering from a COVID-19 infection (11). Nevertheless, a recent study shows that the time interval following infection does not affect IVF/ICSI outcomes (20). Unfortunately, the sample size in the above three studies is relatively small to provide validated evidence. Previous studies with smaller sample sizes have shown mixed results regarding the optimal timing of IVF post-COVID-19. Our larger cohort study provides more robust evidence that immediate initiation of IVF treatment post-recovery is feasible and safe. No trend or dose-response relationship was observed across intervals (≤60 to >180 days), suggesting oocyte quality and pregnancy rates are stable regardless of recovery time. This supports initiating IVF immediately post-recovery without waiting for a “cooling-off” period, countering earlier suggestions of delaying treatment for 3 months (11). Moreover, the isolated difference in blastocyst formation rates (**Table 5**) lacked clinical correlation (e.g., no pregnancy rate differences) and may reflect random variation given multiple comparisons.

### Strength and limitations

CThe strengths of our study include its large sample size, prospective design, and comprehensive assessment of both oocyte and embryo quality and pregnancy outcomes. The consistency of our results across a large cohort strengthens the generalizability of the conclusion that COVID-19 does not meaningfully impair IVF outcomes, addressing inconsistencies in prior smaller studies. Besides, we rigorously adjusted for confounders (e.g., age, BMI, ovarian reserve) in multivariate analyses, which smaller studies often lack the power to do. This confirms the independence of the observed association between COVID-19 and degenerated oocytes. More importantly, our stratified analysis across four post-infection intervals (up to >180 days) is the most comprehensive to date, demonstrating that IVF timing post-recovery is unlikely to affect outcomes. This directly informs clinical practice by alleviating concerns about treatment delays. However, there are limitations. While the total cohort (n=1,169) provides robust power for primary outcomes, subgroup analyses—particularly for pregnancy outcomes in fresh transfers (n=328) and time intervals (e.g., n=72 for ≤60 days)—were underpowered to detect subtle effects. Additionally, long-term pregnancy outcomes and neonatal health were not assessed in this study, which are important aspects for future research.

## Conclusions

In conclusion, our prospective cohort study demonstrates that prior COVID-19 infection is associated with a modest increase in the number of degenerated oocytes but does not negatively impact embryo quality or pregnancy outcomes. Furthermore, the timing of IVF treatment following COVID-19 recovery was not associated with changes in reproductive outcomes, suggesting that delaying treatment post-infection may be unnecessary. These findings provide reassurance to both patients and clinicians planning assisted reproduction after recovery from COVID-19.

Our results align with recent large-scale studies, such as that by Huang et al. (20), confirming that prior infection does not compromise IVF success. By using a prospective design, comprehensive time-interval stratification, and rigorous adjustment for confounders, our study further supports the resilience of assisted reproductive outcomes post-COVID-19.

Notably, emerging evidence from related research highlights the importance of monitoring other COVID-19-associated reproductive risks. For instance, a recent study by Ma et al. (2025) found an association between COVID-19 vaccination and increased risk of pregnancy-induced hypertension in women undergoing assisted reproduction (27). However, vaccination, especially using mRNA-based vaccines, seems to be very safe in women undergoing IVF/ICSI as analyzed by Chen et al. (2022) (28). While our study did not evaluate vaccination status or hypertensive disorders, these findings underscore the need for continued surveillance and mechanistic exploration of COVID–19–related effects, whether from infection or vaccination, on reproductive health.

Future research should focus on long-term outcomes such as live birth, neonatal health, and possible immunologic or vascular sequelae in pregnancies conceived post-COVID-19. This broader understanding will be essential for guiding evidence-based fertility care in the evolving landscape of post-pandemic reproductive medicine.

## Data Availability

The raw data supporting the conclusions of this article will be made available by the authors, without undue reservation.
