# Supplementary figures and images for: Prior COVID-19 infection increases degenerated oocytes but does not affect IVF outcomes: a prospective cohort study
Source: Front Endocrinol (Lausanne). 2025 May 22;16:1599771. doi: 10.3389/fendo.2025.1599771 (PMC12137096; doi:10.3389/fendo.2025.1599771)

Supplementary materials

Supplementary figure S1. Flow chart.


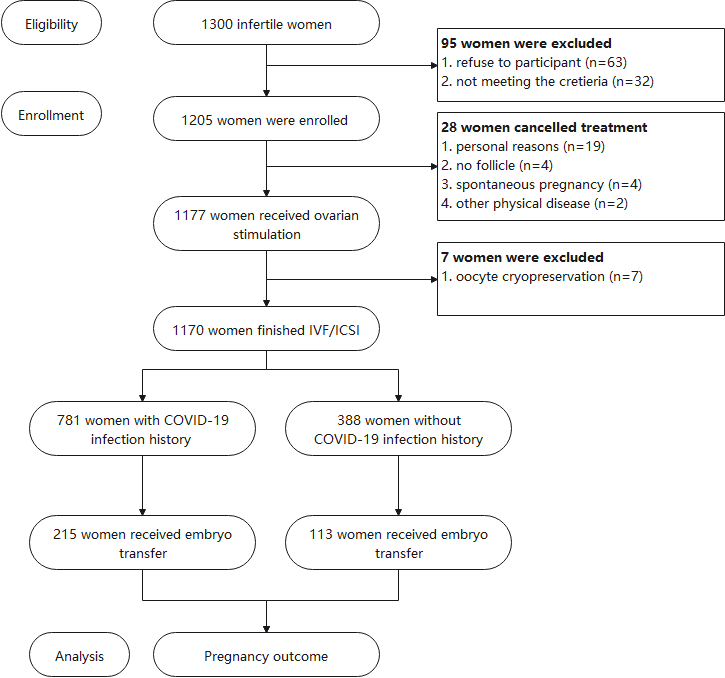

Supplement: Supplementary file 1 [file DataSheet1.docx]
